# Cervical clear cell carcinoma: Case report and literature review

**DOI:** 10.1097/MD.0000000000037449

**Published:** 2024-03-29

**Authors:** Dongying Su, Xia Song, Fang Wu, Shufeng Fan, Miaoer Li

**Affiliations:** a Department of Radiology, The Second Affiliated Hospital of Zhejiang Chinese Medical University, Hangzhou, China.

**Keywords:** Case report, cervix, clear cell carcinoma, diagnosis, imaging

## Abstract

**Rationale:**

Clear cell carcinoma (CCC) is a highly invasive malignant tumor. CCCs of the female reproductive system occur mostly in the endometrium and ovaries and rarely in the cervix. So, it is difficult to diagnose cervical clear cell carcinoma (CCAC) on imaging. This report helps to further deepen our understanding of CCAC.

**Patient concerns:**

A 39-year-old female patient presented with vaginal discharge with no obvious cause, elevated levels of carcinoembryonic antigen (CEA), CA125, CA153, and squamous cell carcinoma antigen (SCC), and underwent ultrasonography (US) CT and MRI examination in our hospital, which showed a mass in the cervix of the uterus, considered of cervical squamous carcinoma.

**Diagnoses:**

The cervix biopsy guided by vaginoscope biopsy and immunohistochemistry confirmed CCAC, combined Magnetic Resonance Imaging examination, CCAC with pelvic lymph node metastasis was considered.

**Interventions and outcomes:**

The patient refused further treatment and was discharged from hospital.

**Lessons:**

CCAC exhibited no specific symptoms, and is slightly different from cervical squamous carcinoma in image features, mainly relying on immunohistochemistry for diagnosis. The reported case raised awareness of CCAC.

## 1. Introduction

Most cervical carcinomas are squamous tumors, followed by adenocarcinomas.^[1]^ CCAC is a rare type of cervical carcinoma with a low morbidity. The pathogenesis of cervical CCAC remains unclearly. A previous study^[2]^ showed that CCAC develops from cervical endometriosis. However, differs from the ovary, whose endometriosis usually presents as a cystic or cystic solid on imaging, CCACs usually present as a soft tissue mass. The boundary of CCACs is usually clearly, which is easily confused with benign lesions. CCACs are highly malignant and prone to lymphatic spread and metastasis, so early diagnosis and treatment of CCAC are essential.

This report describes a case of CCAC. Clinical manifestations, clinicopathological features, diagnosis, and treatment have been discussed, along with a literature review. This case finding provides a more widespread understanding for radiologists in the diagnosis of cervical CCC.

## 2. Case description

**Figure 1.**
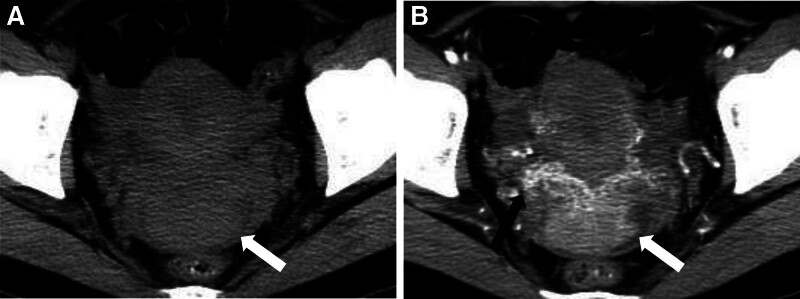
CT image showing clear cell adenocarcinoma of the uterine cervix. (A) Tumor with soft tissue density and a clear boundary (white arrow). The arterial phase image (B) shows obvious enhancement of the tumor (white arrow). Thickened uterine arteries and veins were observed around the bottom of the uterus (black arrow). The venous phase image (C) shows continuous enhancement and (D) enlarged lymph nodes around the bilateral iliac vessels (white arrow).

**Figure 2.**
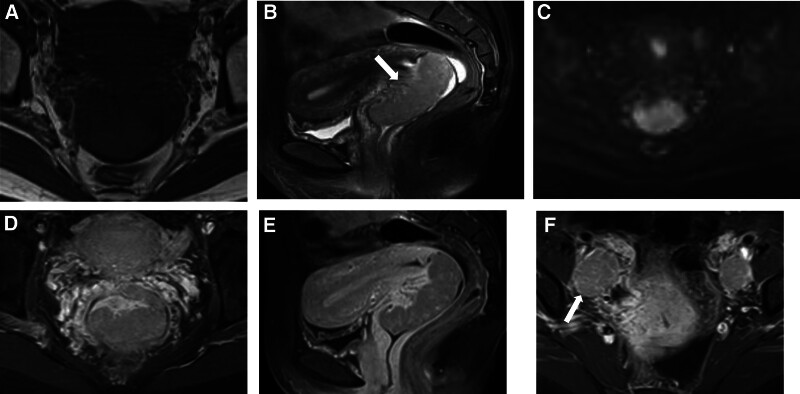
MRI image showing clear cell adenocarcinoma of the uterine cervix. Axial T1 weighted MR image (A) shows a lesion in the uterine cervix, with a slightly hyperintense signal. Sagittal T2 fat suppression image (B) shows hyperintense signal intensity and uniform cystic change of tumor (white arrow). Diffusion-weighted magnetic resonance imaging (C) shows limited diffusion in the tumor. Axial (D) and sagittal enhanced images (E) show significant tumor enhancement. Axial-enhanced image (F) shows enlarged lymph nodes around the bilateral iliac vessels (white arrow).

**Figure 3.**
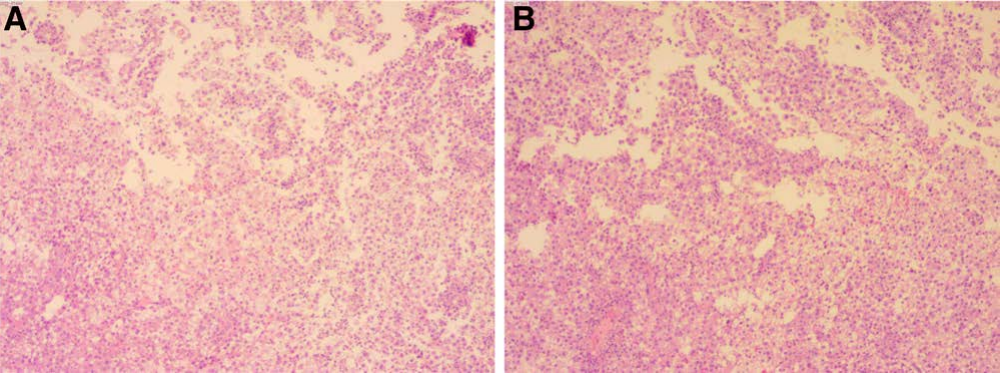
Microscopic examination with high power (A) shows clear tumor cells. The cytoplasm is bright and part of which is eosinophilic.

A 39-year-old woman presented to our hospital with vaginal discharge for more than 1 month, which had a light yellow color and a strong odor. The patient had no history of diethylstilbestrol exposure. Laboratory examinations revealed the following: elevated levels of carcinoembryonic antigen (CEA), CA125, CA153, and squamous cell carcinoma antigen (SCC); normal levels of other serum tumor markers; and a negative human papillomavirus (HPV) test result. Ultrasound (US) showed an isoechoic lump in the cervix of the uterus, protruding into the vagina. Its boundary was clear, and its size was approximately 5.4 × 2.9 × 4.4 cm. Color Doppler Flow Imaging showed abundant blood flow signals, and hypoechoic nodules were observed in the bilateral accessory areas, which were considered lymph nodes. Enhanced abdominal computed tomography (CT) and pelvic magnetic resonance imaging (MRI) were performed. Enhanced CT showed that the cervix was enlarged with a soft tissue density lump, whose boundary was clear (Fig. 1). The lesions showed uniform and obvious enhancement in the arterial phase of enhanced scanning, continuous enhancement in the venous phase, and decreased enhancement in the delayed phase. Tortuous and enlarged uterine arteries and veins were observed at the lower uterus, with the lesions receiving blood supply from the uterine arteries. Multiple enlarged lymph nodes were observed around the bilateral iliac vessels and the abdominal aorta, and the degree and mode of enhancement were similar to those of the cervical lesions. Pelvic MRI revealed an enlarged uterus, with an oval-like solid mass on the anterior wall of the cervix, approximately 6.3 × 2.6 cm in size. The mass, which was protruding into the upper vagina, compressed and thinned the anterior wall of the cervix. The boundary between the mass and the muscular layer of the anterior wall of the cervix was unclear, the interface was not smooth, and the continuity of the local muscular layer was not good; however, the boundary between the mass and the posterior wall of the cervix was clear, with it protruding into the vagina. Compared with the myometrium, the mass showed a slightly hyperintense signal on T1-weighted imaging and fat-suppressed T2-weighted imaging, with several marked hyperintense signals on fat-suppressed T2-weighted imaging in the lesions. Hyperintense signals were observed on diffusion-weighted imaging (*b* = 800) with a decreased apparent diffusion coefficient (0.992 × 10^−3^ mm^2^/s). On enhanced MRI, the mass showed obvious enhancement, which was lower than that of the uterine muscle layer. The uterine body and bilateral appendages had not been invaded. Only a small amount of fluid accumulation was seen in the uterine cavity, and multiple enlarged lymph nodes were observed beside the iliac vessels bilaterally, with hyperintense signals apparent on diffusion-weighted imaging (*b* = 800) and a decreased apparent diffusion coefficient (0.864 × 10^−3^ mm^2^/s). On enhanced MRI, the lymph nodes showed obvious enhancement, similar to that of the cervical lesion. A small amount of liquid was observed in the pelvic cavity (Fig. 2). A malignant cervical tumor with lymph node metastasis was suggested according to clinical and imaging findings. To confirm the pathology of the cervical tumor, a biopsy of the cervical mass was performed. Macroscopic examination showed gray and white broken tissue. The pathologic findings were solid and flaky tumor cells that were medium-sized and slightly atypical, with clear cell boundaries, round and deeply stained nuclei, and a transparent and partially acidophilic cytoplasm. Immunohistochemical analysis showed P16(+), CK+, CK10(focal+), P53(+), ER(−), PR(−), PAX-8(−), HMB45(−), S-100(−), KI67(40%+) (Fig. 3). On the basis of the pathological and immunohistochemical results, CCC of the cervix was considered. On enhanced abdominal CT and pelvic MRI, no obvious abnormalities were found in the other abdominal organs, endometrium, or ovaries. So, the patient was diagnosed with a primary CCC of the cervix. Therefore, further surgical treatment was suggested, however, the patient refused.

## 3. Discussion

Most cervical carcinomas are squamous tumors, followed by adenocarcinomas. Adenocarcinomas are histologically categorized into mucinous, endometrioid, clear cell, serous, and mesonephric subtypes. Clear cell adenocarcinoma of the uterine cervix is rare, accounting for only 4% to 9% of all cervical adenocarcinomas.^[3]^ CCC, which occurs in the female reproductive system, originates in the accessory mesonephric duct. It is composed of cells with a transparent cytoplasm and unique morphological characteristics. Most of these occur in the endometrium and ovary, and rarely in the cervix. A previous study^[4]^ found that CCC occurs in different parts of the uterus and exhibits similar histological and biological characteristics.

Cervical CCC is a highly invasive malignant tumor. Clinically, CCAC can be asymptomatic or present with atypical abnormal vaginal bleeding or vaginal discharge, which was atypical.^[5]^ A previous study^[4]^ reported a bimodal age distribution with peaks at median ages of 26 and 71 years; the age of the patient was 39 years. CCC has characteristic clear tumor cells that contain glycogen-containing cells with abundant clear cytoplasm and hobnail cells. Sasaki et al^[6,7]^ believed that clear tumor cells are characterized by rich transparent cytoplasm and round nuclei located in the center or periphery, no prominent nucleoli, rich in glycogen, protruding cell membranes, deep staining of nuclei, and low mitotic rate.^[8]^ Histologically, CCC of the cervix should be considered when glycogen-containing clear hobnail cells are found. Immunohistochemically, ER, PR, HNFlB, napsin A, and P504s are effective markers for differentiating CCCs.^[9]^

However, the pathogenesis of cervical CCAC remains unknown. Most studies have shown that its pathogenesis is unrelated to HPV infection.^[8,10]^ Previous studies^[10]^ on endometrial CCC showed that endometrial CCC is associated with exposure to diethylstilbestrol in utero. Stolnicu et al^[11]^ showed that CCCs may develop from cervical endometriosis or tuboendometrioid metaplasia. The patient tested negative for HPV and had no history of DES exposure.

Currently, there are few reports on the imaging findings of CCAC. Most CCACs are endophytic and tend to exhibit deep cervical infiltration, creating a barrel-shaped cervix.^[4]^ Most CCACs show expansive growth and clear boundaries. CCC is a tumor with a rich blood supply, and the tumor parenchyma is rich in sinusoidal blood vessels, showing obvious enhancement. This case showed a solid mass with a clear boundary in the cervix, which showed a hyperintense signal on T1-weighted imaging and fat-suppressed T2-weighted imaging compared to the myometrium, after which it was obviously enhanced. The degree of enhancement was lower than that of the myometrium, which was similar to a previous study.^[12,13]^ We believe that the slightly hyperintense signal intensity of CCAC on plain scan T1-weighted imaging is due to CCAC being composed of cells with an accumulation of dissolved lipids and cholesterol.^[14]^ A previous study^[14]^ reported that cytoplasmic fat was detected in up to 60% of CCC cases. Another study^[15]^ suggested that because CCCs arise from endometriotic cysts, these signal intensities might be attributable to the degenerated blood products of endometriotic cysts. The boundary between the lesion and the muscular layer of the anterior wall is unclear, which indicates the invasion of the muscular layer of the anterior wall.^[13]^ Ovarian CCC usually presents as a cystic or cystic solid, which may be due to the fact that ovarian CCC occurs on the basis of an ovarian endometriotic cyst. Our case of CCAC showed soft tissue mass. This may be explained by the limited growth of the endometrial cysts in the cervix. In the cervix, the fibrous connective tissue surrounding the cyst restricts its growth. Thus, the solid components increased and formed a solid mass.^[16]^

CCAC is prone to lymphatic spread and metastasis, which often involves the internal and external iliac regions, particularly the parauterine and pelvic regions.^[4,17]^ In this patient, multiple enlarged lymph nodes were observed near the bilateral iliac vessels and the abdominal aorta and lymph node metastasis was considered. Immunohistochemical staining of Ki67 40% + Ki67 is an immunohistochemical index reflecting the proliferation of tumor cells, and the expression level of Ki67 is closely related to lymph node metastasis in cervical cancer.^[13]^

Differential diagnoses of CCAC include cervical squamous cell carcinoma, cervical polyps, and cervical submucosal myoma. Cervical squamous cell carcinoma usually appears as a soft tissue mass surrounding the cervical canal with unclear boundaries. Our case was limited to the downward growth of the anterior cervical wall with a clear boundary. Cervical polyps and submucosal myomas were benign. In this case, diffusion-weighted images showed limited diffusion and a thickened uterine artery blood supply could be seen on an enhanced scan, which is usually a sign of malignant tumors. CCAC is associated with a high degree of malignancy, poor prognosis, metastasis, and recurrence. Surgery in combination with chemotherapy is an effective treatment.^[18]^ CT and MRI are not only helpful in predicting the histopathological features of CCC of the cervix before surgery but also contribute to preoperative staging.

In summary, the morphology of CCAC is different from that of CCC of the ovary and endometrium and is difficult to diagnose using pathology and immunohistochemistry alone. The final diagnosis must be based on imaging, pathology, and immunohistochemistry. Surgery combined with chemotherapy is an optimal treatment option.

## Author contributions

**Conceptualization:** Dongying Su, Miaoer Li.

**Writing—original draft:** Dongying Su, Miaoer Li.

**Data curation:** Xia Song, Fang Wu.

**Supervision:** Shufeng Fan, Miaoer Li.

**Writing—review & editing:** Shufeng Fan, Miaoer Li.
